# Response: Commentary: Azathioprine as an adjuvant therapy in severe Graves’ disease: a randomized controlled open-label clinical trial

**DOI:** 10.3389/fendo.2024.1473224

**Published:** 2024-12-20

**Authors:** Magdy Mohamed Allam, Ramy Mohamed Ghazy, Hanaa Tarek Hussein Elzawawy

**Affiliations:** ^1^ Internal Medicine Department, Alexandria University Student Hospital (AUSH), Alexandria, Egypt; ^2^ Tropical Health Department, High Institute of Public Health Alexandria University, Alexandria, Egypt; ^3^ Family and Community Medicine Department, College of Medicine, King Khalid University, Abha, Saudi Arabia; ^4^ Internal Medicine Department, Faculty of Medicine, Alexandria University, Alexandria, Egypt

**Keywords:** azathioprine, hyperthyroidism, remission, Graves', severe

We appreciate Mittal et al.’s interest in our study (1) and would like to address the points they raised in their commentary. Our study demonstrates that azathioprine (AZA), when used as an adjuvant therapy, significantly improves remission rates and reduces relapse in patients with severe, refractory Graves’ disease (GD). These findings suggest that immunosuppressive therapy may play a crucial role in improving outcomes for patients who do not respond adequately to conventional antithyroid treatments, with potential implications for evolving treatment protocols in clinical practice.

In our research, participants were randomly allocated using a computer-generated sequence with a 1:1:1 allocation ratio, stratified by baseline thyroid function. The study included 270 patients participants (90 in each group) (range 30-65 years). Gender distribution was similar across groups, with 60% female in the AZA group and 58% in the control group. Baseline thyroid function and duration of disease were comparable across groups, minimizing potential confounding effects. We randomized untreated hyperthyroid patients with severe GD into three groups. All patients received the conventional therapy based on American Thyroid Association guidelines including 45-mg carbimazole (CM) as the starting dose and propranolol 40–120 mg daily. The first and second groups received an additional AZA 1 mg/kg/day, and 2 mg/kg/day, respectively).

Our findings indicate that AZA, as an adjuvant therapy, was effective in reducing disease severity and relapse rates. Notably, our study showed a significantly higher remission rate in the AZA group compared to the control group (87.5% vs. 33.4%, p = 0.002). This finding is consistent with previous research indicating that early immunosuppressive treatment with AZA can significantly decrease the frequency of GD complications and thyrotoxicosis recurrence (2, 3). Additionally, the decline in FT4, FT3, and TSH receptor antibodies (TRab) concentrations was faster in the AZA2 group, further supporting the efficacy of AZA in managing GD.

Although this study provides valuable insights into the use of AZA as an adjuvant therapy, several limitations should be considered. The relatively small sample size may limit the generalizability of the results. Moreover, the open-label design could introduce potential bias. Future studies with larger sample sizes, double-blind designs, and fixed dosing regimens will be necessary to confirm these findings and refine treatment recommendations.

Our findings suggest that AZA may be a beneficial addition to treatment protocols for patients with severe and refractory GD, particularly those who fail to achieve remission with conventional antithyroid therapy. By enhancing remission rates and reducing relapse risk, azathioprine offers a promising alternative for managing difficult-to-treat cases. As personalized medicine advances, identifying patients who would benefit most from immunosuppressive therapy could further optimize outcomes. Future research should focus on integrating AZA into standardized treatment algorithms, especially in cases resistant to conventional approaches

## Addressing criticisms and concerns

### Criticism: inconsistencies in remission criteria

The identification of remission cases as defined in these references ranges from a simple control of symptoms passing to a simple control of function according to the definition of the ATA (4), so we differentiate between two types of remission. The stringent criteria we used to define remission, including normalization of thyroid hormone levels and resolution of hyperthyroid symptoms for a continuous period of at least 12 months without antithyroid medication, may have contributed to the lower observed remission rates. These strict criteria ensure a robust assessment of long-term disease control but may result in lower remission rates than studies with less rigorous definitions.

### Criticism: low remission rate among patients receiving conventional therapy with ATD

Regarding the concern related to the unusually low remission rate among patients receiving conventional therapy with ATD. We assessed remission in severe cases, not all GD. Our study focused specifically on patients with severe and refractory GD, a subset that is typically more challenging to treat and may not respond as well to conventional therapies. This patient population is expected to have lower remission rates than those with less severe forms of the disease. Although our remission rates may appear lower than those reported in other studies, it is important to consider differences in study populations, remission definitions, and duration of follow-up. Our findings are consistent with other research focusing on similarly severe cases of GD, where conventional ATD therapy alone is often insufficient. Adherence to medication and intensity of monitoring can significantly affect remission rates. In our study, we implemented strict adherence monitoring protocols. However, real-world factors, such as variations in adherence and follow-up intervals, could also influence remission results. We suggest that future studies should further investigate the factors contributing to the variability in remission rates, such as genetic predispositions, environmental influences, and differences in access and quality of healthcare. Additionally, larger, multicenter, and different severity of GD trials could provide more comprehensive data on remission rates in diverse patient populations.

### Criticism: conflicting findings were observed with different doses of azathioprine used in the study

We appreciate the feedback regarding the use of different doses AZA and the resulting conflicting findings. The doses of AZA used in the study were chosen based on existing literature and clinical practice guidelines for autoimmune diseases (5). We aimed to balance efficacy with safety, avoiding excessively high doses that could lead to adverse effects while ensuring sufficient immunosuppressive action. We closely monitored patients for adverse effects and adjusted doses as needed to ensure safety. Patients received AZA within a specified dose range, which allowed for adjustments based on individual tolerance and response. Moreover, the conflicting findings with different doses of AZA may reflect individual variability in drug metabolism and response. Some patients may have achieved remission with lower doses, while others required higher doses for similar outcomes. These variations underscore the importance of personalized medicine in treating complex conditions like severe GD. Finally, the study’s open-label design allowed for real-time dose adjustments, which contributed to variations in dosing but was crucial for patient safety and treatment efficacy. Future studies should focus on identifying biomarkers that predict response to AZA, which could help tailor dosing more precisely. Additionally, controlled trials with fixed dosing regimens could provide clearer insights into the optimal dosing strategies for AZA in severe GD.

### Criticisms of lack of clarity regarding follow-up duration

The study was designed with a follow-up period of 12 months after stopping the medication followed by 12 months after remission. The duration of treatment is observed for 18 months according to the ATA guidelines. This duration was selected to balance the need for sufficient time to observe the effects of AZA as an adjuvant therapy with the practical constraints of conducting a long-term clinical trial. The patients were monitored at regular intervals throughout the follow-up period. These intervals included quarterly evaluations to evaluate thyroid function, remission status, and any adverse effects. The primary endpoint of the study was the remission status at the end of the 12-month follow-up period. Secondary endpoints included the duration of remission, the time to relapse, and the incidence of adverse effects throughout follow-up. All follow-up assessments were performed using standardized protocols to ensure consistency and reliability in data collection. We acknowledge that individual patient needs and responses may have led to some variability in follow-up duration for specific cases. However, the core follow-up period was consistently applied across the study population. Future studies could benefit from longer follow-up periods to further assess the long-term efficacy and safety of AZA as an adjuvant therapy in severe GD. Additionally, establishing clearer guidelines for follow-up protocols would enhance the comparability of results across different studies.

### Criticism: regarding discussion and conclusion

Our study demonstrated that AZA, when used as an adjuvant therapy, showed potential in reducing disease severity and relapse rates in patients with severe GD who did not respond adequately to conventional anti thyroid medications therapy. These results indicate that immunosuppressive therapy could play a beneficial role in managing refractory cases. We employed Kaplan-Meier survival analysis, log-rank testing, and Fisher’s exact test for categorical data comparison to analyze the data. These methods were chosen based on their appropriateness for evaluating time-to-event outcomes and exploring associations between variables of interest. While proportional hazards analysis (Cox regression) or nominal logistic regression can provide additional insights by controlling for multiple confounding factors simultaneously, However, our initial analysis adjusted for key variables such as age, gender, baseline thyroid function, and duration of disease. We acknowledge the importance of considering a broader range of potential confounders, such as treatment adherence, comorbidities, and genetic factors, in further multivariate models. All these study results were discussed in detail in the discussion section and highlighted in the conclusion to provide a comprehensive overview of the findings.
